# Pharmacological management of metabolic syndrome and its lipid complications

**Published:** 2010

**Authors:** T. Binesh Marvasti, Kh. Adeli

**Affiliations:** Clinical Biochemistry, Research Institute, The Hospital for Sick Children, University of Toronto, Toronto, Ontario

**Keywords:** HDL-cholesterol, LDL Cholesterol, Triglycerides (TG), Type 2 diabetes

## Abstract

Obesity epidemic has been spread all over the world in the past few decades and has caused a major public health concern due to its increasing global prevalence. Obese individuals are at higher risks of developing dyslipidemic characteristics resulting in increased triglyceride and LDL-cholesterol content and reduced HDL-cholesterol levels. This disorder has profound implications as afflicted individuals have been demonstrated to be at increased risk of development of hypertension, atherosclerosis, type 2 diabetes and cardiovascular diseases. Today, this phenotype is designated as metabolic syndrome. According to the criteria set by the International Diabetes Federation (IDF), for a patient to be diagnosed with metabolic syndrome, the person must have central obesity plus any two of the following conditions: raised TG, reduced HDL-cholesterol, raised blood pressure, and increased fasting plasma glucose. Current National Cholesterol Education Program Adult Treatment Panel III (NCEP-ATP III) guidelines for the treatment of patients with the metabolic syndrome encourage therapies that lower LDL cholesterol and TG and raise HDL-cholesterol. Primary intervention often involves treatment with statins to improve the lipid profiles of these patients. However, recent studies suggest the potential of newly identified drugs including thiazolidinediones, GLP-1 agonists, and DPP-4 inhibitors that seem to be promising in reducing the level of progression of metabolic syndrome related disorders. This review discusses the current pharmacological treatments of the metabolic syndrome with the above mentioned drugs.

## I. Metabolic Syndrome

Metabolic syndrome, also known as the insulin resistance syndrome, is a cluster of correlated metabolic abnormalities including glucose intolerance (elevated fasting glucose), insulin resistance, obesity, dyslipidemia (reduced HDL cholesterol, elevated plasma triglycerides (TG), and increased LDL cholesterol), and hypertension (1, 2).

According to the International Diabetes Federation (IDF), for a patient to be diagnosed with metabolic syndrome, the person must have central obesity (defined by waist circumference with ethnicity specific values) plus any two of the following conditions: raised TG, reduced HDL cholesterol, raised blood pressure, and increased fasting plasma glucose (3). Metabolic syndrome has emerged in recent years as a major public health concern due to its increasing global prevalence. This disorder has profound implications as afflicted individuals have been demonstrated to be at increased risk of development of hypertension, atherosclerosis, type 2 diabetes and cardiovascular disease (2).

Prevalence of metabolic syndrome varies among different populations depending on gender, age, and ethnicity (4). Results from a population study in Prevention of Metabolic Disorders Research Center in Tehran, Iran indicate that among 4018 Iranian subjects 40 years and older, the prevalence of metabolic syndrome was 51.4% (5) hence putting Iranian populations at high risk of complications associated with this syndrome (i.e. cardiovascular diseases).

Clinical repercussions of the metabolic syndrome include alterations in glucose and lipid homeostasis of which insensitivity to the actions of insulin is a key feature. This ‘resistance’ to insulin action is thought to promote a dyslipidemic state in which hepatic gluconeogenesis, glucose output, and VLDL secretion are enhanced. Furthermore, normal post- prandial suppression of adipose tissue lipolysis is compromised in the insulin resistant state leading to a persistent elevation in circulating free fatty acid (FFA) levels. Enhanced FFA mobilization triggers a variety of metabolic deficiencies including further decreases in insulin sensitivity and subsequent development of hyperinsulinemia (6). Current National Cholesterol Education Program Adult Treatment Panel III (NCEP-ATP III) guidelines for the treatment of patients with the metabolic syndrome encourage therapies that lower LDL cholesterol and TG and raise HDL cholesterol (1). Primary intervention often involves treatment with statins to improve the lipid profiles of these patients.

The NCEP-ATP III criteria for the metabolic syndrome are based on the presence of 3 or more of the followings: increased waist circumference, elevated triglyceride levels, blood pressure (BP), fasting glucose level, and reduced HDL-C levels. The American Heart Association (AHA) and National Heart, Lung and Blood Institute (NHLBI) groups affirm the overall utility and validity of the NCEP-ATP III criteria and proposed that they should continue to be used with modifications. The modifications include: 1-adjustment of waist circumference to lower thresholds when individuals or ethnic groups are prone to insulin resistance,

2-allowing TG and HDL-C levels and BP to be counted as abnormal when a person is prescribed drug treatment for these conditions, 3-clarifying that elevated BP is defined as an elevation of either systolic or diastolic BP, and 4-reducing the threshold for elevated glucose level from 110 mg per dL or higher to 100 mg per dL or higher.

The International Diabetes Federation (IDF) has proposed clinical criteria similar to those of the NCEP-ATP III with identical thresholds for TG and HDL-C levels, BP, and plasma glucose. The IDF criteria are different in that the waist circumference thresholds are adjusted to different ethnic group and gender and require that increased waist circumference to be considered as an element of the metabolic syndrome because abdominal obesity reflects both concepts of obesity and insulin resistance (7). In the U.S. population, updated NCEP-ATP III and IDF criteria identify essentially the same people as having the metabolic syndrome (7).

Recommendations for management of the metabolic syndrome are for the most part the updated NCEP- ATP III and IDF reports. First-line recommendations for reducing Cardiovascular Heart Disease (CHD) risks include smoking cessation, reducing LDL-C levels, BP, and glucose levels to recommended goals. If the TG level is higher than 500 mg per dL, then lowering the TG level to 500 mg per dL or less takes primacy over LDL-C lowering. After LDL-C, TG, and non HDL-C goals are achieved, a fourth target is raising HDL-C level. No specific goals for raising HDL-C levels are specified. Lifestyle interventions include weight loss in obese subjects, increased physical activity, and dietary modification.

LDL-lowering standard drugs are also recommended. These include statins and ezetimibe. Other newly identified drugs that reduce the level of progression of metabolic syndrome related disorders include thiazolidinediones, GLP-1 agonists, and DPP-4 inhibitors. In this review, current pharmacological treatment of the metabolic syndrome and its lipid complications with the above mentioned drugs will be discussed.

## II. Statins: Effective cholesterol lowering agents with multiple pleiotropic effects

Statins are a class of drugs that in their active hydrolysed form are specific inhibitors of HMG-CoA reductase, the enzyme responsible for catalyzing the conversion of HMG-CoA to mevalonate, an early rate limiting step in the cholesterol biosynthesis pathway. Inhibition of HMG-CoA reductase with statins has been shown to reduce the plasma levels of cholesterol and apoB-containing lipoproteins in hypercholesterolemic models (8). This is achieved through increased hepatic cholesterol uptake due to upregulation of the LDL receptor as well as mechanisms of action on apoB, such as reduced translocation across the endoplasmic reticulum (ER) membrane, increased intracellular degradation, and diminished lipoprotein assembly (9).

Statins have been shown to influence the secretion of both LDL and VLDL in patients with hyperlipidemia (10). More recently, statins were also found to be effective in reducing plasma TG levels, inhibiting intracellular cholesterol biosynthesis and upregulating LDL receptor expression (11). As a result of many years of study, statins have been characterized as highly effective hypolipidemic agents to reduce lipid abnormalities in metabolic syndrome patients (12). Some of the most common statins currently in clinical use are as follows.

### II-1. Atorvastatin

As a member of the statin family, atorvastatin has been clinically shown to lower LDL-C levels and recent studies also show that it can also ameliorate hepatic lipoprotein overproduction (13).

In a clinical study examining the effect of atorvastatin, subjects with type 2 diabetes mellitus and dyslipidaemia (LDL-C >100 mg/dl in those without coronary artery disease, n = 77; LDL-C >70 mg/dl in those with coronary artery disease, n = 4) were initiated with 10 mg of atorvastatin daily. The mean body mass index among men and women subjects was 25.0 +/- 4 and 26.7 +/- 3.6 kg/m2 respectively. Pretreatment mean HbA(1c) was 7.9 +/- 1.8% and total cholesterol, TG and HDL-C and LDL-C were 214 +/- 27 mg/dl, 164 +/- 63 mg/dl, 46 +/- 6 mg/dl and 135 +/- 24 mg/dl respectively. After three months of treatment the mean decrease was 62 +/- 31 mg/dl in total cholesterol (p < 0.001), 31 +/- 57 mg/dl in TG (p < 0.001), 51 +/- 27 mg/dl in LDL-C (p < 0.001) and 4 +/- 8 mg/dl in HDL-C (p < 0.001). The LDL-C level was reduced by 37.6% in these patients, from 135 +/- 24 mg/dl to 84 = 27 mg/dl (p < 0.001) with 10 mg of atorvastatin daily. It was possible to achieve target LDL-C of less than 100 mg/dl in 75.5% (n = 58) in subjects without CHD (n = 77) and less than 70 mg/dl in 75% (n = 3) of those patients with CHD (n = 4). The present study showed that in patients with type 2 diabetes mellitus, 10 mg of atorvastatin daily was safe, well tolerated, and effective in reducing LDL-C to target levels (14). Our laboratory has examined the mechanism of action of atorvastatin in animal models of insulin resistance such as the fructose-fed hamster. In a series of *ex vivo* experiments in fructose-fed Syrian golden hamsters, atorvastatin treatment was shown to block hepatic apoB overproduction induced by the development of insulin resistance. In this study, there was molecular evidence of improved hepatic insulin sensitivity with atorvastatin treatment based on assessment of the phosphorylation status of the insulin receptor and the expression of protein tyrosine phosphatase- 1B. The improvement in insulin signalling was not mediated by a change in hepatic TG accumulation as no significant difference was observed in liver TG levels. Taken together, these data suggested that atorvastatin not only can reduce the LDL-C levels, but it may potentially contribute to enhanced hepatic insulin sensitivity.

### II.2. Simvastatin

Clinical studies have recently suggested that statin treatment may beneficially elevate plasma concentrations of HDL cholesterol in patients with hyperlipidemia and increase the anti-atherogenic involvement of this cholesterol in the blood vessels (15). Elevated plasma LDL cholesterol levels and low levels of HDL cholesterol are known risk factors for development and progression of atherosclerosis and coronary heart disease (16).

Epidemiological evidence has demonstrated that elevated plasma levels of apoA1, the major HDL apolipoprotein, is a strong negative risk factor for the development of coronary heart disease (17). Normal regulation of plasma HDL concentration is directed in part by the rate of synthesis and secretion of apoA1 from hepatocytes. In a study conducted in our laboratory, simvastatin treatment was shown to acutely increase synthesis and secretion of apoA1 in both hepatoma cell line (HepG2) and primary hepatocyte cultures derived from Syrian golden hamsters (15). This statin treatment caused the greatest increase in apoA1 levels at 10 µM as compared with control cells. Higher doses of simvastatin did not have a further stimulatory effect on apoA1 levels (15).

Additionally in order, to determine whether the stimulatory effect of simvastatin on apoA1 is a classical effect of statins in general or specific to this drug, the effect of simvastatin on intracellular synthesis of apoA1was compared to the effect of lovastatin in both HepG2 and primary hamster hepatocytes. The differential effect of both statins on HepG2 cell production of apoA1 in a dose response experiment showed that both statins exhibit a dose dependent effect on apoA1 synthesis and the greatest effect was observed following a 10µM treatment. However, simvastatin exhibited a more potent ability to increase apoA1 synthesis (44.3 ± 12.1%, 10 µM) in comparison with lovastatin (26 ± 2%, 10µM). These data indicate the specificity of the observed statin effects (15).

Clinical studies have also shown that simvastatin can potentially raise plasma HDL concentrations by as much as 17%. This beneficial effect together with reduced plasma LDL-cholesterol concentration are accompanied by significant reductions in the risk of morbidity in patients with coronary heart disease (42%) relative to patients receiving standard care (18).

### II.3. Rosuvastatin

Rosuvastatin is a new member of the statin family with higher efficacy in reducing LDL cholesterol than other statins at comparable doses (19) by having a higher number of binding interactions with HMG-CoA reductase, compared to other statins (20, 21). This enhanced binding may cause stronger inhibition of the enzyme and hence result in a greater therapeutic efficacy. As well, rosuvastatin has a longer half-time than other statins and a higher degree of selectivity for hepatic cells (the main site of cholesterol synthesis) compared to non-hepatic cells (22).

In a study conducted by Park et.al, an open-labeled, randomized trial was performed to compare the effects of 10 mg of rosuvastatin and 10 mg of atorvastatin on lipid and glycemic control in Korean patients with nondiabetic metabolic syndrome (23). In total, 351 patients who met the modified NCEP- ATP III criteria for metabolic syndrome with low- density lipoprotein cholesterol (LDL-C) levels ≥ 130 mg/dL were randomized to receive either rosuvastatin 10 mg (n = 173) or atorvastatin 10 mg (n = 178) for over 6 weeks.

After 6 weeks of treatment, greater reductions in total cholesterol (- 35.94 ± 11.38 vs.-30.07 ± 10.46%, *p* < 0.001), LDL-C (48.04 ± 14.45 vs. 39.52 ± 14.42%, *p* < 0.001), non-high-density lipoprotein cholesterol (- 42.93 ± 13.15 vs.-35.52 ± 11.76%, *p* < 0.001), and apolipoprotein-B (- 38.7 ± 18.85 vs.-32.57 ± 17.56%, *p* = 0.002) levels were observed in the rosuvastatin group as compared to the atorvastatin group. Overall, the percentage of patients attaining the NCEP ATP III goal was higher with rosuvastatin as compared to atorvastatin (87.64 vs. 69.88%, *p* < 0.001). Hence, Rosuvastatin 10 mg was more effective than atorvastatin 10 mg in achieving NCEP ATP III LDL-C goals in patients with nondiabetic metabolic syndrome, especially in those with lower NCEP ATP III target level goals (23).

## III. Cholesterol Absorption Inhibitors

Cholesterol absorption inhibitors are a class of medication that decrease the absorption of cholesterol in the small intestine and hence decrease its delivery to the liver hence allowing more cholesterol to be cleared from the blood. The intestine has a unique capability to act as a gatekeeper for entry of cholesterol into the body, and inhibition of intestinal cholesterol absorption is now widely regarded as an attractive non-statin therapeutic strategy for dyslipidemia prevention. Here one of the most widely used subclasses of this group of cholesterol lowering medications, ezetimibe will be descussed.

**Table 1:** Current and emerging pharmacological treatments for the metabolic syndrome

Drug Famil:	Name, Functions and Applications:
**Statins**	**a. Atorvastatin**
	-Lowers LDL-C levels
	-Ameliorates hepatic lipoprotein overproduction
	-Enhances hepatic insulin sensitivity and signaling
	**b. Simvastatin**
	-Lowers LDL-C levels
	-Increases plasma concentrations of HDL-C through elevating plasma concentration of apoA1 in patients with hyperlipidemia
	**c. Rosuvastatin**
	-Has the highest efficacy in reducing LDL-C, apoB, and non HDL-C than other statins at comparable doses

**Cholesterol Absorption Inhibitors**	**a. Ezetimibe**
	-Inhibits the intestinal absorption of dietary and biliary cholesterol
	-Reduces the LDL-C levels by 20–25% when administered as either monotherapy or in combination with a statin
	-Reduces C-reactive protein levels

**Insulin sensitizing agents**	**a. Rosiglitazone**
	-Ameliorates whole body insulin resistance
	-Improves hepatic insulin signaling
	-Reduces hepatic lipoprotein over production
	**b. Metformin**
	-Reduces excessive rates of hepatic glucose production
	-Improves insulin-stimulated glucose utilization by extra hepatic tissues
	-Lowers circulating levels of plasminogen activator inhibitor-1, hence lowering atherothrombotic events
	-Reduces progression to diabetes among overweight and obese glucose-intolerant individuals
	-Decreases weight gain in part by reducing dietary energy intake
	-A useful adjunct in insulin-requiring patients with type 2 diabetes

**GLP-1 Receptor Agonists**	**a. Exenatide**
	-Stimulates replication and inhibits apoptosis of islet beta cells
	-Sustains reductions in HbA1c levels with progressive loss of body weight
	-Improves glycemic control as a result of progressive reductions in body weight
	-Improves blood pressure, lipid profiles and hepatic transaminase levels
	-Reduces the infiltration of fat into liver

**DPP-4 Inhibitors**	**a. Sitagliptin**
	-Used either alone or in combination with other oral antihyperglycemic agents (such as metformin or a thiazolidinedione) for treatment of diabetes mellitus type 2
	-Improves glycaemic control in adults with type 2 diabetes
	-Not licensed for use in combination with insulin therapy
	-Significantly reduces HbA1c and fasting plasma glucose levels
	-Ameliorates insulin secretion through improving the proinsulin to insulin ratio

### III. 1. Ezetimibe

Ezetimibe is a member of a novel class of lipid lowering agents known as cholesterol absorption inhibitors (24). Ezetimibe inhibits the intestinal absorption of dietary and biliary cholesterol by binding to the Niemann- Pick C1 Like 1 (NPC1L1) transport protein (25). At the FDA approved dose of 10 mg/day, ezetimibe has been shown to provide a 20–25% reduction in LDL cholesterol levels when administered as either monotherapy or in combination with a statin (21). It is recommend that when standard doses of statins are insufficient to achieve the goal LDL cholesterol of less than 100 mg/dL, either the statin dose should be increased or ezetimibe may be prescribed alongside the statins (26). Despite the absence of data evaluating the impact of ezetimibe on the progression of atherosclerosis in metabolic syndrome and cardiovascular disease patients, the number of prescriptions dispensed for ezetimibe to be taken alone or in combination with simvastatin has increased substantially from 2003 to 2006 in the USA (19).

In April 2008, the ENHANCE (Ezetimibe and Simvastatin in Hypercholesterolemia Enhances Atherosclerosis Regression) group conducted a study on the clinical utility of ezetimibe in hypercholesterolemic patients at risk for atherosclerosis (24). In this study, 720 patients with hypercholesterolemia were randomized and treated with a combination of 10 mg/day of ezetimibe plus 80 mg/day of simvastatin over two years of follow-up and the results showed reduced LDL cholesterol and C-reactive protein levels to a significantly greater degree with this combination than 80 mg/day of simvastatin monotherapy (p < 0.01). However, despite these differences in cholesterol-lowering, no significant difference in the change in carotid intima media thickness, a measure of subclinical atherosclerosis and the primary endpoint of the study, was observed in either treatment group. The ENHANCE trial later raised important questions regarding whether ezetimibe impacts biological processes relevant to the pathogenesis of atherosclerosis in addition to lipid-lowering.

Statins have been shown to improve endothelial mediated vasodilatory responses through increased nitric oxide bioavailability, and has potent anti- inflammatory, antioxidant, and antithrombotic properties (27). These effects likely contribute to the positive impact of statins on clinical outcomes that cannot be accounted for by reduction in LDL cholesterol alone (27). The impact of ezetimibe on these processes, which are integral to the pathogenesis of atherosclerosis, remains unclear. Landmesser et al.^28^ randomized 20 patients with New York Heart Association (NYHA) class III chronic heart failure and a mean LDL cholesterol of 106 mg/dL to 4 weeks of 10 mg/day of simvastatin or 10 mg/day of ezetimibe monotherapy and evaluated the impact on endothelial function. They observed that simvastatin and ezetimibe combination treatment yielded equivalent reductions in LDL cholesterol levels in comparison with simvastatin monotherapy. This finding enabled them to compare the pleiotropic effects of each therapy directly. Endothelial function was assessed by radial artery ultrasound flow-mediated dilation (FMD), a noninvasive measure that quantifies the percent change in radial artery diameter in response to reactive hyperemia. Compared with baseline, mean ± SEM FMD significantly increased after 4 weeks of simvastatin treatment (from 5.1% ± 0.7% to 10.5% ± 0.6%; p <0.01), whereas no significant change was observed with ezetimibe (from 5.8% ± 0.6% to 5.6% ± 0.5%). Interestingly, the improvement in FMD after simvastatin was paralleled by an increase in endothelium-bound extracellular superoxide dismutase (ecSOD) activity, a vascular antioxidant enzyme, whereas no significant change in ecSOD activity was observed with ezetimibe. These data suggest that the improvement in endothelial function conferred by simvastatin may have been mediated, at least in part, by an antioxidant mechanism. The number of functionally active endothelial progenitor cells (EPCs) isolated from peripheral blood mononuclear cells was also significantly increased by simvastatin (p < 0.05), but not by ezetimibe. While some studies suggest no improvement in the endothelial mediated vasodilatory response with ezetimibe treatment, other studies suggest the opposite. Ezetimibe therapy has yielded a positive effect on endothelial function in trials including patients with metabolic syndrome. First, an open- label pilot study was conducted in 14 men with metabolic syndrome who were on 40 mg/day of atorvastatin (29). Endothelial function was assessed by venous plethysmography after intrabrachial artery acetylcholine infusion at baseline and 8 weeks after a therapeutic switch to 10 mg/day of atorvastatin and 10 mg/day of ezetimibe. Although the reduction in LDL cholesterol (21.0%) was not significantly different compared with baseline, total cholesterol (16.5%), VLDL cholesterol (32.2%), and TG (34.9%) were significantly lower after 8 weeks of treatment. However, due to reductions in total cholesterol and lack of a parallel comparison with statin monotherapy, the independent impact of ezetimibe on endothelial function in this population was difficult to ascertain.

Another prospective, randomized, crossover study evaluated the impact of 80 mg/day of simvastatin and the combination of 10 mg/day ezetimibe and 10 mg/day of simvastatin on endothelial function in 19 metabolic syndrome patients without a history of cardiovascular disease (30). After 6 weeks of treatment, brachial artery FMD was measured before and 4 hrs after ingestion of an oral fat load (50 g fat/m2 and 3.75 g glucose/m2). Despite similar reductions in total cholesterol and LDL over 6 weeks (mean ± SE 7.6% ± 1.2% to 7.7% ± 1.6%; p = 0.8), the combination of ezetimibe and low- dose simvastatin, attenuated the acute reduction in endothelial function elicited by the oral fat load. These conflicting results are not conclusive and more research is required to be conducted on the effects of ezetimibe on endothelial function in metabolic syndrome.

## IV. Insulin Sensitizers and PPAR γ agonists

Currently, insulin sensitizing drugs, such as metformin and thiazolidinediones, are part of the treatment of practically any type 2 diabetic patient and can also be used in patients with the metabolic syndrome. At the beginning of the therapy, metformin is often used; later, insulin sensitizers (PPAR-gamma stimulators) such as of rosiglitazone are also prescribed. The relevance of PPARgamma (peroxisome- proliferator-activated receptor gamma) as an important therapeutic target for the treatment of diabetes arises from its hypoglycaemic effects in diabetic patients and also from the critical role in the regulation of cardiovascular functions.

### IV.1. Insulin sensitizing agents: Rosiglitazone and Metformin

Clinical studies on type 2 diabetes patients indicate that the combination therapy of rosiglitazone and metformin have the highest therapeutic effects. In a randomized double-blind, controlled trials undertaken in clinics in Canadian centres, 207 patients with impaired glucose tolerance were randomly assigned to receive combination of rosiglitazone (2 mg) and metformin (500 mg) twice daily or matching placebo for a median of 3.9 years. Incident diabetes occurred in significantly fewer individuals in the active treatment group (n = 14 [14%]) than in the placebo group (n = 41 [39%]; p < 0.0001). The relative risk reduction was 66% and the absolute risk reduction was 26% yielding a number needed to treat of 70 (80%) patients in the treatment group. Insulin sensitivity decreased by the end of study in the placebo group and remained unchanged with rosiglitazone and metformin treatment (p = 0.0006 between groups) (31).

Postprandial elevation of TG-rich lipoproteins (TRLs) is a well-recognized feature of diabetic dyslipidemia and includes the accumulation of intestinally derived apolipoprotein B48 (apoB48)-containing lipoproteins (8). There is increasing evidence that TRLs, including the apoB48 intestinally derived lipoproteins, may be particularly atherogenic (32). To date, studies in rats and human have focused on the delayed clearance of TRLs as the dominant underlying mechanism for their postprandial accumulation in diabetes. The impaired removal of intestinally derived lipoproteins has been attributed to decrease in the activity of lipoprotein lipase (33) and apolipoprotein composition of the carrier particles (34).

Rosiglitazone treatment of the fructose-fed hamster has been shown to ameliorate whole body insulin resistance, improve hepatic insulin signalling, and reduces hepatic lipoprotein over production (35, 36). Treatment of fructose-fed hamsters with rosiglitazone resulted in an approximately 50% lower apoB48 secretion rate than fructose alone (p < 0.05), but treatment of chow fed hamsters with rosiglitazone resulted in no significant change in apoB48 secretion rate.

The principal action of metformin (dimethyl- biguanide) is to reduce excessive rates of hepatic glucose production and improve insulin-stimulated glucose utilization by extrahepatic tissues.

Metformin targets the enzyme adenosine 5'mono- phosphate activated protein kinase (AMPK) (37, 38). AMPK regulates cellular glucose and lipid metabolism through phosphorylation of the main proteins that affect energy production. Metformin lowers circulating levels of plasminogen activator inhibitor-1, which may contribute to fewer atherothrombotic events (38). In the Diabetes Prevention Program, metformin has been shown to reduce progression to diabetes among overweight and obese glucose-intolerant individuals (39). It may decrease weight gain in part by reducing dietary energy intake, and is widely regarded as a useful adjunct in insulin-requiring patients with type 2 diabetes.

## V. Drugs acting on the Incretin System

A major and progressive defect in type 2 diabetes is the relative reduction in the ability of glucose to trigger insulin secretion from beta cells (38). Enhancing insulin secretion through a glucose dependent mechanism has been considered an attractive therapeutic approach since it can restore the defective physiological pathway in type 2 diabetes patients and minimize the risk of hypoglycaemia.

Gastrointestinal polypeptide hormones that are secreted in response to ingestion of a meal augment postprandial insulin secretion. This is known as the incretin effect and can account for up to 70% of postprandial insulin secretion (40). Quantitatively, the two most important incretin hormones are glucagon-like peptide-1 (GLP-1) and glucose dependent insulinotropic peptide (GIP). These hormones are secreted by the L cells of the distal ileum and colon, and the K cells of duodenum and upper jejunum respectively. Circulating concentrations of incretins rise within a few minutes of eating, implying likely stimulation via activation of neuroendocrine pathways. Incretins act via specific G-protein coupled receptors on beta cells to enhance glucose-stimulated insulin secretion (41). The acute effect of GLP-1 on beta cells is stimulation of glucose- dependent insulin release, followed by enhancement of insulin biosynthesis and stimulation of insulin gene transcription.

The incretin effect is almost completely deficient in patients with type 2 diabetes mainly because of reduced postprandial GLP-1 secretion that is accompanied by reduced action of GIP (42).

Administration of GLP-1 to individuals with type 2 diabetes increases insulin secretion and produces a sustained dose dependent reduction in plasma glucose levels in concert with a reduction in body weight (43). GLP-1 and GIP are subject to rapid degradation mainly by the ubiquitous cell surface enzyme dipeptidyl peptidase-4 (DPP-4), which cleaves two N-terminal amino acids thereby removing insulinotropic activity. Anti -diabetic DPP-4 inhibitors reduce serum activity of DPP-4 by 80% after a single dose through irreversible inhibition of the enzyme.

Incretin mimetics fall into two categories: (i) derivatives of GLP-1 modified to resist proteolysis; and (ii) novel peptides that have metabolic action similar to GLP-1 and are intrinsically resistant to proteolysis (44).

### V.1. Glucagon-Like Peptide-1 Receptor Agonists

#### V.1.1 Exenatide

A DPP-4-resistant agonist for the GLP-1 receptor, exendin-4, was first isolated from the venom of the Gila monster (Heloderma suspectum), a lizard native to the south-western US and Mexico, in a systematic search for new therapeutic peptides [41]. Synthetic exendin-4, known as exenatide, was approved in 2005 by the FDA and has subsequently been licensed in many other countries (38). Exenatide is delivered by twice-daily subcutaneous injections using a prefilled pen device. The drug is licensed as adjunctive therapy for patients with type 2 diabetes with inadequate glycaemic control who are taking metformin, a sulfonylurea, a thiazolidinedione, a combination of metformin and a sulfonylurea, or a combination of metformin and a thiazolidinedione. As a GLP-1 receptor agonist, exenatide stimulates replication and inhibits apoptosis of islet beta cells (45) as well as improving first-phase insulin secretion (46).

Sustained reductions in HbA1c levels with progressive loss of body weight have been demonstrated during long term treatment with exenatide. A systematic review and meta-analysis trial of at least 12 weeks duration concluded that the GLP-1 receptor agonist lowered glycated haemoglobin compared with placebo (weighted mean difference −0.97%) and induced weight loss (1.4 kg and 4.8 kg for placebo and insulin respectively). Further studies have shown that improvements in glycemic control are sustained with progressive reductions in body weight (47). Glycaemic control and reduced body weight may be accompanied by improvements in blood pressure, lipid profiles and hepatic transaminase levels suggesting a reduction in the infiltration of fat into liver (48).

### V.2. Dipeptidyl Peptidase-4 Inhibitors

Pharmaceutical inhibitors of DPP-4 are agents taken orally that acutely increase postprandial plasma levels of endogenous GLP-1 typically by two fold. The primary target of these drugs are postprandial hyperglycaemia, although fasting plasma glucose levels are also reduced. In clinical trials, HbA1c reductions have ranged from 0.5–1.1% in monotherapy when these agents have been administered at therapeutic doses once or twice daily. Greater reductions have been observed in patients with higher baseline HbA1c levels (49).

#### V.2.1. Sitagliptin

Prescription of sitagliptin (100 mg once daily) was approved in the USA in 2006 and in Europe in April 2007 (49). As a DPP-4 inhibitor, sitagliptin is indicated as an adjunct to diet and exercise modifications to improve glycaemic control in adults with type 2 diabetes. Sitagliptin is not licensed for use in combination with insulin therapy.

In a 18 week study of sitagliptin monotherapy, 521 adults with type 2 diabetes treated with diet and exercise with a mean baseline HbA1c of 8.1% were randomized to treatment with placebo, or100 mg or 200 mg of sitagliptin once daily. HbA1c was significantly reduced (−0.6%) with 100 mg daily of sitagliptin compared with placebo; 200 mg daily of sitagliptin was no more effective than 100 mg daily. Fasting proinsulin to insulin ratio were significantly improved with sitagliptin treatment while the incidence of hypoglycaemia and gastrointestinal symptoms was not significantly different between sitagliptin and placebo groups. The efficacy and safety of sitagliptin, added to ongoing metformin therapy, were assessed in 701 patients with type 2 diabetes who had inadequate glycaemic control (HbA1c ≥7% and ≤10%; mean 8.0%) with metformin alone. Mean duration of diabetes in these patients was 6–6.5 years and mean BMI 31 kg/m2. After a 2-week, single-blind, placebo run in period, patients receiving ongoing metformin (> = 1500 mg/day) were randomly assigned to receive placebo or 100 mg of sitagliptin once-daily in a 1: 2 ratio for 24 weeks. At week 24, the addition of sitagliptin led to significant reductions in HbA1c (−0.65%), fasting plasma glucose and 2-hour post-meal glucose concentrations. Various measures of β-cell function, including proinsulin to insulin ratio were significantly improved with sitagliptin compared with placebo.

## Concluding Remarks

The metabolic syndrome is a constellation of common metabolic disorders that is associated with type 2 diabetes and cardiovascular disease. Insulin resistance and dyslipidemia play central roles in the pathophysiology of this syndrome. Patients diagnosed with metabolic syndrome often exhibit raised TG and LDL cholesterol, reduced HDL cholesterol and raised blood pressure and fasting plasma glucose (3). Classical medications for the treatment of metabolic syndrome include LDL cholesterol lowering agents such as the statins. However, recent studies show that increasing insulin sensitization by PPAR γ agonists (i.e. rosiglitazone) or augmenting postprandial insulin secretion using gastrointestinal polypeptide hormones can also ameliorate the complications caused by this syndrome. Examples of these recently developed medications have been discussed in this review. Intense research is currently underway to develop new therapeutic strategies in the treatment and management of this very common and clinical important syndrome.
